# Diazonium Salt-Based Surface-Enhanced Raman Spectroscopy Nanosensor: Detection and Quantitation of Aromatic Hydrocarbons in Water Samples

**DOI:** 10.3390/s17061198

**Published:** 2017-05-24

**Authors:** Inga Tijunelyte, Stéphanie Betelu, Jonathan Moreau, Ioannis Ignatiadis, Catherine Berho, Nathalie Lidgi-Guigui, Erwann Guénin, Catalina David, Sébastien Vergnole, Emmanuel Rinnert, Marc Lamy de la Chapelle

**Affiliations:** 1 CSPBAT Laboratory, UMR 7244, UFR SMBH, University of Paris 13, Sorbonne Paris Cite, 93017 Bobigny, France; inga.tijunelyte@univ-paris13.fr (I.T.); nathalie.lidgi-guigui@univ-paris13.fr (N.L.-G.); 2 BRGM, F-45060 Orléans CEDEX 02, France; i.ignatiadis@brgm.fr (I.I.); C.Berho@brgm.fr (C.B.); 3 IFREMER, Brittany Center, Detection, Sensors and Measurements Laboratory, CS10070, 29280 Plouzané, France; Jonathan.Moreau@ifremer.fr (J.M.); Emmanuel.Rinnert@ifremer.fr (E.R.); 4 Laboratoire TIMR, EA4297, Sorbonne Universités—Université de Technologie de Compiègne, Centre de recherche de Royallieu, rue du docteur Schweitzer, CS 60319, 60203 Compiègne CEDEX, France; erwann.guenin@utc.fr; 5 HORIBA Jobin Yvon SAS, 59650 Villeneuve d’Ascq, France; Catalina.DAVID@horiba.com (C.D.); sebastien.vergnole@horiba.com (S.V.)

**Keywords:** polynuclear aromatic hydrocarbon (PAH), surface-enhanced Raman spectroscopy (SERS), nanosensor, diazonium salt, surface functionalization, detection

## Abstract

Here, we present a surface-enhanced Raman spectroscopy (SERS) nanosensor for environmental pollutants detection. This study was conducted on three polycyclic aromatic hydrocarbons (PAHs): benzo[a]pyrene (BaP), fluoranthene (FL), and naphthalene (NAP). SERS substrates were chemically functionalized using 4-dodecyl benzenediazonium-tetrafluoroborate and SERS analyses were conducted to detect the pollutants alone and in mixtures. Compounds were first measured in water-methanol (9:1 volume ratio) samples. Investigation on solutions containing concentrations ranging from 10^−6^ g L^−1^ to 10^−3^ g L^−1^ provided data to plot calibration curves and to determine the performance of the sensor. The calculated limit of detection (LOD) was 0.026 mg L^−1^ (10^−7^ mol L^−1^) for BaP, 0.064 mg L^−1^ (3.2 × 10^−7^ mol L^−1^) for FL, and 3.94 mg L^−1^ (3.1 × 10^−5^ mol L^−1^) for NAP, respectively. The correlation between the calculated LOD values and the octanol-water partition coefficient (K_ow_) of the investigated PAHs suggests that the developed nanosensor is particularly suitable for detecting highly non-polar PAH compounds. Measurements conducted on a mixture of the three analytes (i) demonstrated the ability of the developed technology to detect and identify the three analytes in the mixture; (ii) provided the exact quantitation of pollutants in a mixture. Moreover, we optimized the surface regeneration step for the nanosensor.

## 1. Introduction

As part of the latest European Water Framework Directives (Directives 2000/60/EC, 2006/118/EC, and 2006/11/EC), the development of analytical tools allowing on-site, accurate, and sensitive detection of pollutants in environmental waters is of primary importance. Although extensive efforts have been devoted to developing highly sensitive, reproducible, accurate, and robust analytical sensors for on-site or in situ monitoring of trace elements (i.e., qualitative and quantitative analysis) [1,2,3,4,5,6,7], organic contaminants have received less attention.

Among the strategies investigated to meet the need for detecting organic contaminants in water, novel extraction techniques have been widely reported. Solid phase microextraction (SPME) [8,9,10,11] or stir-bar sorptive extraction (SBSE) [12] combined with gas chromatography (GC) [13,14,15], high performance liquid chromatography (HPLC) [15,16], mass spectrometry (MS) [17], Raman spectroscopy [10], or capillary electrophoresis (CE) have led to accurate results down to pg L^−1^ concentrations for chlorinated solvents [16], benzene, toluene, ethylbenzene, and xylenes (BTEXs) [10], and polycyclic aromatic hydrocarbons (PAHs) [9,15]. Despite their high sensitivity, procedures using these passive sensors are ill-suited for on-site monitoring due to their limitations regarding the co-injection of solvents [18,19] and the desorption procedure under high temperature and/or pressure [20]. 

New alternative methodologies with similar efficiencies are thus needed for detecting organic contaminants. Given their low limit of detection on the order of ng L^−1^, piezoelectric chemical sensors (quartz crystal microbalance, QCM) [21,22,23,24,25,26,27,28,29,30,31] have attracted considerable attention. Although QCM is suitable for investigating the preconcentration of organic contaminants using coated polymers or calixarenes [22,23,24,25,26,27,28,29,31,32], the use of QCM-based sensors for the detection of various organic compounds in natural samples has not been reported as far as we know, probably because they cannot identify individual compounds.

In comparison, issues related to compound identification in complex environmental conditions can be overcome by exploiting surface-enhanced Raman spectroscopy (SERS). SERS is a powerful technique based on nanostructured metallic surfaces that greatly enhance the Raman signal via both electromagnetic and chemical effects. The first effect arises from the interaction between the incident light and the metallic nanostructures, inducing local enhancement of the electromagnetic field through the excitation of localized surface plasmons [33,34,35,36,37]. As a result, the Raman signal of any molecule located in close vicinity to the nanostructured surface can be enhanced up to 10^8^-fold [38,39,40,41,42]. The second effect contributing to Raman signal enhancement by up to 10^2^-fold is due to the electronic interaction between the molecules and the metallic nanostructures (i.e., charge transfer between surface and chemisorbed molecules) [43]. SERS nanosensors can detect very low concentrations of analytes and thus, attain high sensitivity and low limit of detection (LOD) values, down to the individual molecule [44,45,46]. Most importantly, SERS provides a molecular fingerprint, thus molecules can be individually identified and deciphered in complex mixtures [47,48].

Finally, due to the improvements in Raman spectrometer miniaturization [11], the innovations in nanoscale technologies applied to sensors (essentially based on colloid systems [49,50]), and the implemented surface chemistry strategies (i.e., surface functionalization by using Self-Assembled Monolayers (SAMs) for analyte preconcentration), SERS has already been recognized as a powerful tool for on-site monitoring of organic contaminants at the ng L^−1^ level [50,51,52,53,54,55,56,57]. However, despite great improvements made to produce colloidal nanostructures with controlled sizes and shapes [49,50], SERS nanosensors have a major drawback related to poor SERS signal reproducibility due to the uncontrollable aggregation of metal colloids. To overcome the lack of reproducibility and capitalize on the increasing number of SERS substrates available commercially, we selected substrates (Wavelet) based on supported gold nanorod arrays produced and distributed by the S.T. Japan company. These SERS substrates are devoted for highly reproducible and very sensitive measurements.

Regarding surface functionalization, SAMs are widely recognized as excellent systems for sensing applications because they offer well-defined organization and densely-packed structures [58]. However, there are limitations with regard to the stability of the bond between the thiol and the metallic surface and hence the stability of the resulting SAMs [59,60]. As a powerful, innovative alternative to SAMs, we adopted a diazonium salt-based surface functionalization strategy. Immobilization of aryldiazonium has been described as a versatile method, providing surface properties that can be fine-tuned given the wide variety of available diazonium salts and stable grafted organic layers [61,62,63,64,65,66,67,68], thereby ensuring sensor robustness. Therefore, we studied a diazonium-salt-functionalized SERS sensor for the detection and the quantitation of three PAHs. Among the 130 PAHs released into the environment, benzo[a]pyrene (BaP), fluoranthene (FL), and naphthalene (NAP) belong to the priority substances under the European Water Framework Directive (2000/60/EC) and the United States Environment Protection Agency. The investigation of selected pollutants was conducted to demonstrate the proof-of-concept of this novel SERS nanosensor for compound sensing. PAH compounds were analyzed alone and in mixture. Investigations of the targeted compounds were carried out with different concentrations prepared in water-methanol (9:1 volume ratio) solutions. Calibration curves and detection limits were established for each compound. Moreover, given that the preconcentration of targeted molecules at the nanosensor surface is essentially based on weak molecular forces, we tested the possibility of regeneration and reuse of the developed nanosensor. 

## 2. Materials and Methods

### 2.1. Reagents

Diethyl ether (≥98%), tetrafluoroboric acid (49.5–50.5%), sodium nitrite (≥97%), 4-dodecylaniline (97%), benzo[a]pyrene (≥96% HPLC), fluoranthene (98%), and naphthalene (99%) were purchased from Sigma-Aldrich and used without further purification. Ethanol (96%) and methanol (100%) were purchased from VWR; sulfuric acid (95–98%) was purchased from JT Baker. Milli-Q water (resistivity of 18.2 MΩ cm^−1^) was used in all experiments.

### 2.2. Diazonium Salt Chemical Synthesis

4-dodecyl benzenediazonium-tetrafluoroborate (DS-C_10_H_21_), was synthesized regarding the reaction between aryl amines and sodium nitrite at 0 °C [69]. Briefly, the primary amine (4 × 10^−3^ mol L^−1^) was dissolved in a tetrafluoroboric acid (HBF_4_) solution and the mixture was then cooled for 15 min. A precooled aqueous sodium nitrite (4.3 × 10^−3^ mol L^−1^) solution was added dropwise to the acid amine mixture under stirring. The mixture was then allowed to react for 40 min. The obtained precipitate was filtered through a 0.2 μm cellulose ester filter (Whatman) and generously washed with milli-Q water. The diazonium salt was purified by re-crystallization in diethyl ether for 48 h at 6 °C. Afterwards, the prepared salt was dried under vacuum and preserved at −20 °C.

The synthesized diazonium salt was characterized by nuclear magnetic resonance (NMR). Spectra were recorded using a Bruker Avance III 400 MHz instrument in a d^6^-DMSO solvent. Tetramethylsilane (TMS) was used as an internal standard. DS-C_10_H_21_:^1^H NMR (400 MHz, DMSO-d^6^): d (ppm) 0.84–0.87 (t, *J* = 6.4 Hz, 3H); 1.25 (s, 14H); 1.61 (s, 2H); 2.81–2.85 (t, *J* = 7.6 Hz, 2H); 7.81–7.83 (d, *J* = 8.4 Hz, 2H); 8.55–8.57 (d, *J* = 8.4 Hz, 2H).

### 2.3. SERS Substrate

Commercially available gold nanorod arrays (Wavelet) (hereafter referred to as gold nanostructures, GNSs) were purchased from S.T. Japan and were used in this study as SERS active substrates.

### 2.4. Surface Functionalization

Chemical (spontaneous) grafting of the diazonium salt was performed by immersing SERS substrates in a 30 mL solution containing the diazonium salt (10^−3^ mol L^−1^) dissolved in H_2_SO_4_ (10^−3^ mol L^−1^). Substrates were then incubated for 12 h at +4 °C. They were then rinsed under mild stirring by immersion in (i) milli-Q water (3 times) and (ii) pure methanol (3 times), for 10 min for each immersion. Afterwards, the functionalized substrates were dried under a mild nitrogen flux.

### 2.5. Solutions of the Targeted Compounds

Benzo[a]pyrene (BaP), fluoranthene (FL), and naphthalene (NAP) were used in this study to test the ability of the diazonium-salt-based SERS sensor to detect the PAH compounds. These PAHs were selected for their different solubility values related to their polarity defined by the octanol-water partition coefficient, K_OW_. The K_OW_ of each compound is summarized in Table 1. 

Due to solubility issues, stock solutions were prepared by dissolving the targeted pollutants in pure methanol (MeOH). Stock solutions of BaP, FL, and NAP were prepared at different concentrations as follows: 10 mg L^−1^ for BaP, 200 mg L^−1^ for FL, and 1000 mg L^−1^ for NAP, and were stored at −20 °C. Stock solutions were diluted daily with milli-Q water (10% of pollutant stock solution in 90% of water % v/v) to obtain stock solutions which were then stored at +4 °C and used for further dilution, keeping a constant volume ratio (9:1) between milli-Q water and MeOH.

### 2.6. Nanosensor Surface Regeneration

**Scheme 1:**
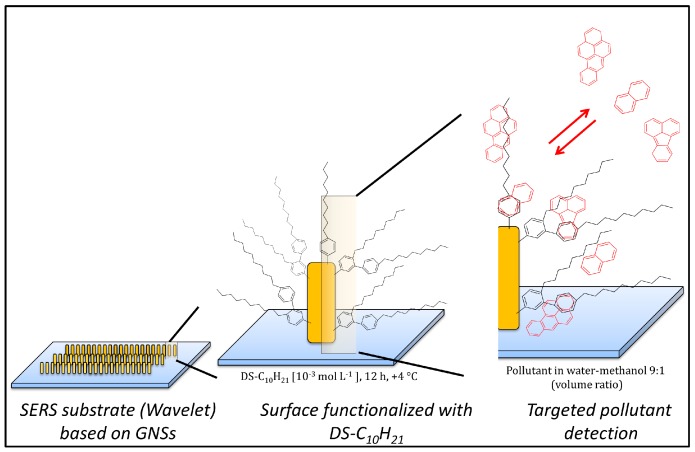
General illustration of nanosensor surface functionalization and preconcentration of the targeted compounds via weak interactions. Gold Nanostructures GNSs, DS-C_10_H_21_, 4-dodecyl benzenediazonium-tetrafluoroborate diazonium salt.

The preconcentration of the targeted pollutants on the SERS nanosensors was driven by weak interactions with the DS-C_10_H_21_ layer, such as hydrophobic interactions and/or π−π stacking (Scheme 1). To regenerate the surface, MeOH was found to be an efficient solution. Thus, after performing the detection of the targeted pollutants, the SERS nanosensors were regenerated with MeOH for 30 min.

### 2.7. Raman and SERS Measurements

Raman and SERS measurements were performed using a transportable micro-Raman spectrometer prototype designed by HORIBA Scientific for on-site applications. The instrumental set-up was equipped with a 691 nm laser diode (Ondax) and a 60× magnification objective (0.7 N.A.) with a collar for glass correction (Olympus). The spectral range was recorded from 400 to 2100 cm^−1^ with a spectral resolution better than 4 cm^−1^. For SERS experiments, the laser power was set to 4 mW to avoid molecular degradation induced by photochemical or thermal effects. The integration time for SERS measurements was set to 20 s with three accumulations to reach a relevant signal-to-noise ratio. Spectral calibration was performed daily on a crystalline silicon sample (peak position at 520 cm^−1^). A fluidic cell located above the microscope objective allowed the sample solutions to flow onto the SERS substrate.

### 2.8. Chemometrics

To remove the Raman and SERS spectral background in a similar way for all spectra, leaving the analytical signal intact, an algorithm was programmed in MatLab 7.0.1 based on [71,72]. Background correction was possible by minimizing the following S function:

(1) $$S=\underset{\left(i\right)}{\sum }\kappa_{i}{\left(y_{i}-z_{i}\right)}^{2}+\lambda \underset{\left(i\right)}{\sum }{\left(\Delta^{2}z_{i}\right)}^{2}$$

where y is the signal intensity for each i wavenumber, z is the baseline, λ is the smoothing parameter, and *p* is the asymmetric parameter as κ_i_ = *p* if y_i_ > z_i_ and κ_i_ = 1 – *p* otherwise. The last term was defined as follows:

(2) $$\Delta^{2}z_{i}=\left(z_{i}-z_{i-1}\right)-\left(z_{i-1}-z_{i-2}\right)$$

The two parameters needed for the calculations, *p* and λ, were set to 10^3^ and 10^−3^, respectively. However for the diazonium-salt-based system during the detection of BaP, the λ value was set to 10^4^ to remove fluorescence induced by impurities. Figure S1 illustrates the background removal process. 

## 3. Results and Discussion

### 3.1. Diazonium Salt Based Surface Functionalization

**Figure 1:**
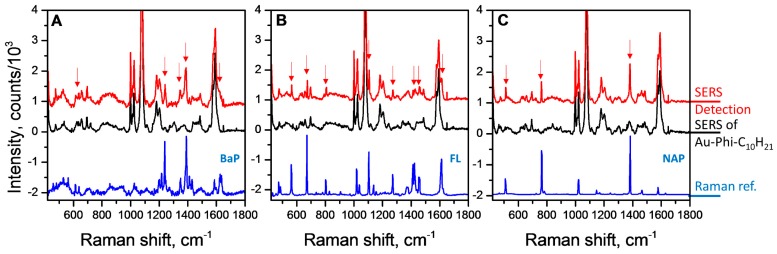
Detection of benzo[a]pyrene (BaP) at 0.75 mg L^−1^ (**A**), fluoranthene (FL) at 5 mg L^−1^ concentration (**B**) and naphthalene (NAP) at 50 mg L^−1^ (**C**) using gold nanostructures (GNSs) functionalized with diazonium salt (Au-Phi-C_10_H_21_). The black and red spectra correspond to the SERS signals before and after incubation in the pollutants solutions, respectively. Blue spectra in (**A**–**C**) correspond to the Raman reference spectra for BaP, FL, and NAP, respectively, acquired on powder. Red and blue spectra are shifted vertically for better visualization. Red arrows indicate the peaks of detected pollutants.

Surface functionalization by aryldiazonium is a convenient method leading to a robust, grafted organic coating. Grafting can be accomplished by either chemical (spontaneous grafting) [65,66,67,68], electrochemical [62,63,64], or photochemical methods [73,74]. A spontaneous grafting strategy was used in this study to functionalize SERS-active substrates. The substrates were immersed in a diazonium salt (DS-C_10_H_21_) solution and incubated at +4 °C for 12 h. The temperature control during the functionalization step is important to drive the covalent grafting and at the same time reduce the rate of spontaneous polymerization [75]. Functionalized substrates were then investigated using SERS. The SERS spectra for DS-C_10_H_21_-GNSs are shown in Figure 1. These spectra are presented in real intensities with the SERS spectral background removed using the algorithm detailed in the Material and Methods section. Because the complete characterization of DS-C_10_H_21_-GNSs can be found in the literature [61], here we just briefly mention that the recorded spectra have a number of features in the spectral ranges that represent the vibrations related to the aromatic ring (see Figure S2 for direct comparison between the SERS spectra of the grafted phenyl derivative and the Raman spectrum of the synthesized diazonium salt). The peak at 1079 cm^−1^ for DS-C_10_H_21_ assigned to the C-H in-plane bending mode coupled with the C-N stretching mode [76] constitutes the signature of the coating. Thus, this peak was used as an internal reference during the quantitation of the detected pollutants. Moreover, this peak does not overlap with the Raman signature of the targeted pollutants (see Figure 1), providing further support for selecting this internal reference.

### 3.2. Detection of PAHs 

Diazonium-salt-based SERS substrates were then evaluated on the sensing of the targeted compounds. Thus, functionalized SERS substrates were incubated in pollutant solutions for 30 min. Investigated concentrations for the initial test were 0.75 mg L^−1^ for BaP, 5 mg L^−1^ for FL, and 50 mg L^−1^ for NAP. After incubation, SERS spectra were measured in six different randomly selected areas on the substrates, covering the entire substrate surface. Averaged spectra for BaP, FL, and NAP detection are shown in Figure 1A–C, respectively. All SERS spectra are presented with removed background.

The successful preconcentration and sensing of the targets by the DS-C_10_H_21_-based SERS sensor was validated after the observation of characteristic fingerprint peaks of each pollutant as summarized in Table 2.

The interaction between the analyte and the coating layer did not induce important shifts in the position of the pollutant peaks. Hence, most of the peak positions were nearly identical to those of the Raman reference spectra. However, in the case of the preconcentration of BaP (Figure 1A), the peak at 1236 cm^−1^ corresponding to the ring stretching and the C-H stretching modes was found shifted 3 cm^−1^ upwards towards higher wavenumbers after adsorption on the nanosensor surface, whereas the peak observed at 1389 cm^−1^ assigned to C-C ring stretching shifted 4 cm^−1^ downwards to lower wavenumbers. Spectral differences mostly related to the vibrational mode intensities were observed for FL (Figure 1B). For instance, the peaks around 1400 cm^−1^ were very weakly enhanced in the SERS spectrum. Such weak spectral modifications may be due to weak interactions between the pollutants and the functionalization layer.

### 3.3. Sensing Performance

After having first proven the ability of the developed DS-C_10_H_21_-based nanosensor to preconcentrate (the targeted) compounds, calibration curves were established to compare the analytical performance of the nanosensor for each pollutant. To do so, the substrate was fixed on the microfluidic cell of the Raman set-up and solutions with increasing concentrations of pollutants were injected onto the substrate at a flow rate of 3 µL min^−1^ for 30 min for each solution.

### 3.4. Benzo[a]pyrene (BaP) Calibration Curve

**Figure 2:**
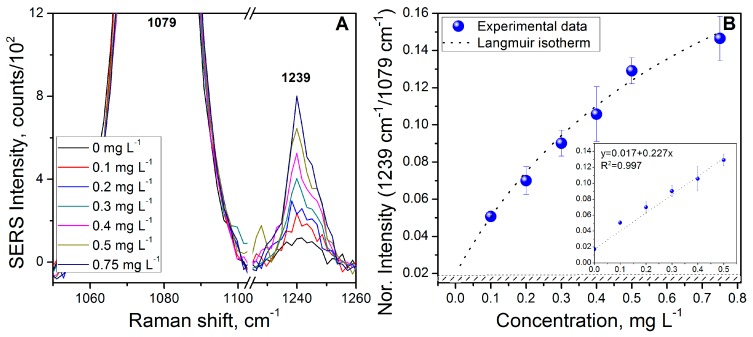
(**A**) Surface-enhanced Raman spectroscopy (SERS) spectra of benzo[a]pyrene (BaP) detection for concentrations from 0 up to 0.75 mg L^−1^; (**B**) calibration curve of BaP detection (blue circles: experimental data, dotted line: Langmuir adsorption isotherm fit). Insert: zoom on the concentration range from 0 to 0.5 mg L^−1^ (blue circles: experimental data, dotted line: linear fit). The hatched area corresponds to the noise level.

BaP solutions with concentrations increasing from 0.1 mg L^−1^ to 0.75 mg L^−1^ (0.4–3.0 µmol L^−1^) were tested to establish the calibration curve. Between each concentration, SERS measurements were carried out on six different areas on the surface. Averaged spectra are shown in Figure 2A, focusing on the peak of BaP at 1239 cm^−1^ (the full spectral range of averaged SERS spectra can be seen in Figure S3). The calibration curve was plotted using the relative intensity of this peak compared with the intensity of the characteristic peak of the coating (i.e., the peak at 1079 cm^−1^) as a function of the analyte concentration (Figure 2B). 

The calibration curve was then fitted using the Langmuir adsorption isotherm expressed as follows:

(3) $$I=\frac{I_{max}Kc}{1+Kc}$$

where I is the normalized SERS intensity, K is the adsorption constant, and c is the analyte concentration.

The calibration plot was found to be linear over the range of 0.1–0.5 mg L^−1^ (0.4–2.0 µmol L^−1^) but surface saturation was not reached. The limiting factor was the solubility of BaP in the milli-Q water-MeOH (9:1 volume ratio) solution.

**Figure 3:**
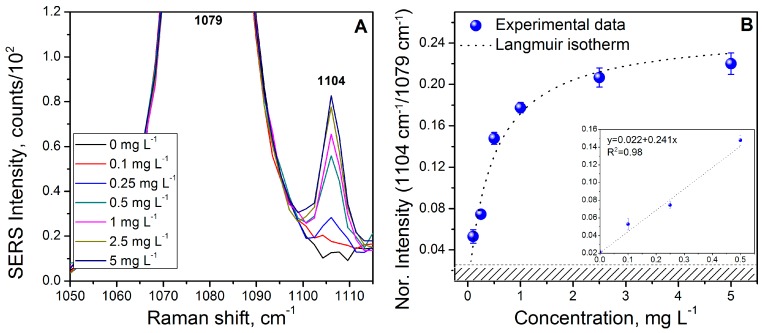
(**A**) Surface-enhanced Raman spectroscopy (SERS) spectra of fluoranthene (FL) detection for concentrations ranging from 0 to 5 mg L^−1^; (**B**) calibration curve of FL detection (blue circles: experimental data, dotted line: Langmuir adsorption isotherm fit). Insert: zoom on the concentration in the range from 0 to 0.5 mg L^−1^ (blue circles: experimental data, dotted line: linear fit). The hatched area corresponds to the noise level.

The slope of the linear regression on the low concentrations (insert in Figure 3B) was used for the calculation of the LOD and the LOQ. LOD is defined as the lowest concentration of analyte in the sample that can be detected [79] and can be calculated using the following formula [80]: LOD = 3 σ/s, where s is the slope of calibration curve and σ is the standard deviation of the blank response. LOQ is the lowest concentration of an analyte that can be determined with acceptable precision and accuracy. LOQ was calculated using the same formula that was used for the calculation of LOD with a confidence level increased to 10: LOQ = 10 σ/s. The calculated LOD and LOQ for BaP were 0.026 mg L^−1^ (0.10 µmol L^−1^) and 0.087 mg L^−1^ (0.34 µmol L^−1^), respectively (Table 3).

### 3.5. Fluoranthene (FL) Calibration Curve

The calibration curve for FL was performed using the same protocol as that for BaP. In comparison with BaP, the investigated concentrations of FL ranged from 0.1 mg L^−1^ to 5 mg L^−1^ (0.5–25 µmol L^−1^) due to its higher solubility (see Table 1). As before, between each concentration, SERS was measured on six different areas on the surface. Averaged spectra are shown in Figure 3A, focusing on the peak at 1104 cm^−1^ (the full spectral range of the averaged SERS spectra is given in Figure S4). Assigned to the FL C-C in-plane stretching mode, this peak was the most intense band observed in the SERS spectrum (Figure 1B). It was thus selected to establish the calibration curve (Figure 3B), in which the relative intensity of this peak compared with the intensity of the peak at 1079 cm^−1^—characteristic of the diazonium salt DS-C_10_H_21_—was plotted as a function of the FL concentration.

At values greater than 3 mg L^−1^, there is a plateau, indicating that the adsorption equilibrium was reached. The calibration curve can be considered linear in the range of 0.1–0.5 mg L^−1^. The LOD of FL was calculated to be 0.064 mg L^−1^ (0.32 µmol L^−1^). The calculated LOQ value was 0.214 mg L^−1^ (1.06 µmol L^−1^).

### 3.6. Naphthalene (NAP) Calibration Curve

**Figure 4:**
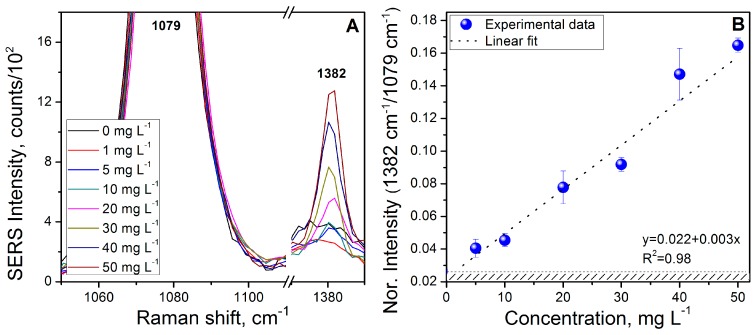
(**A**) Surface-enhanced Raman spectroscopy (SERS) spectra of naphthalene (NAP) detection for concentrations ranging from 0 to 50 mg L^−1^; (**B**) calibration curve of NAP detection (blue circles: experimental data, dotted line: linear fit). The hatched area corresponds to the noise level.

For the study of NAP pre-concentration and detection, SERS measurements were carried out as previously described in milli-Q/MeOH (9:1 v/v) media containing different concentrations of NAP ranging from 1 mg L^−1^ up to 50 mg L^−1^ (7.8–390 µmol L^−1^). NAP peaks observed in the SERS spectra are shown in Figure 1C and summarized in Table 2. Figure 4A focuses on the increase in the intensity of the peak located at 1382 cm^−1^, assigned to the C=C stretching mode (the full spectral range averaged SERS spectra are shown in Figure S5). This peak was chosen to establish the calibration curve shown in Figure 4B since it is the only one that does not overlap with the vibrational signature of the grafted diazonium salt. 

The calibration curve was found to be linear over the investigated range. The calculated LOD and LOQ values were 3.9 mg L^−1^ (30 µmol L^−1^) and 13 mg L^−1^ (0.1 mmol L^−1^), respectively (Table 3).

### 3.7. Analysis in a Mixture of Analytes

**Figure 5:**
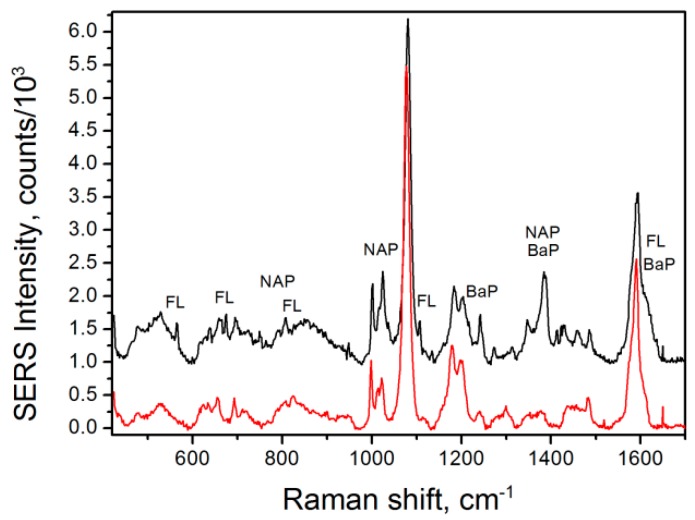
Black spectrum corresponds to the surface-enhanced Raman spectroscopy (SERS) spectrum obtained for the analysis of a mixed solution composed of benzo[a]pyrene BaP (0.75 mg L^−1^, 3 µmol L^−1^), fluoranthene FL (5 mg L^−1^, 25 µmol L^−1^), and naphthalene (NAP) (5 mg L^−1^, 39 µmol L^−1^). The red spectrum shows the SERS signal of the DS-C_10_H_21_ diazonium-salt-based gold nanostructures (GNSs) for comparison. The black spectrum is vertically shifted for clarity.

An experiment using a mixture of the three investigated pollutants was performed to further assess the feasibility of the designed nanosensor for PAHs sensing in water samples and to demonstrate the SERS spectra suitability for on-site screening purposes. For this experiment, a mixed solution of BaP (0.75 mg L^−1^, 3.0 µmol L^−1^), FL (5 mg L^−1^, 25 µmol L^−1^), and NAP (5 mg L^−1^, 39 µmol L^−1^) was prepared in pure water-MeOH (9:1 volume ratio) media. The concentration of BaP was notably lower due to the solubility issue discussed above. The SERS substrate was exposed to the prepared mixture for 30 min before starting SERS measurements. The averaged SERS spectra obtained before and after substrate exposure to the pollutants are shown in Figure 5. At the selected concentrations, BaP and FL were detected and well identified. However, most NAP peaks overlapped either the signal of the coating (i.e., at 1022 cm^−1^) or the signal of the other analytes (i.e., the peak used for establishing the calibration curve for NAP at 1382 cm^−1^ was found combined with the signal of BaP). Nonetheless, it showed one weak, but characteristic peak at 762 cm^−1^. Together, these results demonstrate that the SERS nanosensor can detect the different pollutants in a mixed solution and that we can clearly identify them by using their spectral signatures. However, the quantitation of BaP, FL, and NAP in the mixture is more problematic, as discussed below.

### 3.8. Feasibility of the Nanosensor for Sensing Polycyclic Aromatic Hydrocarbons

**Figure 6:**
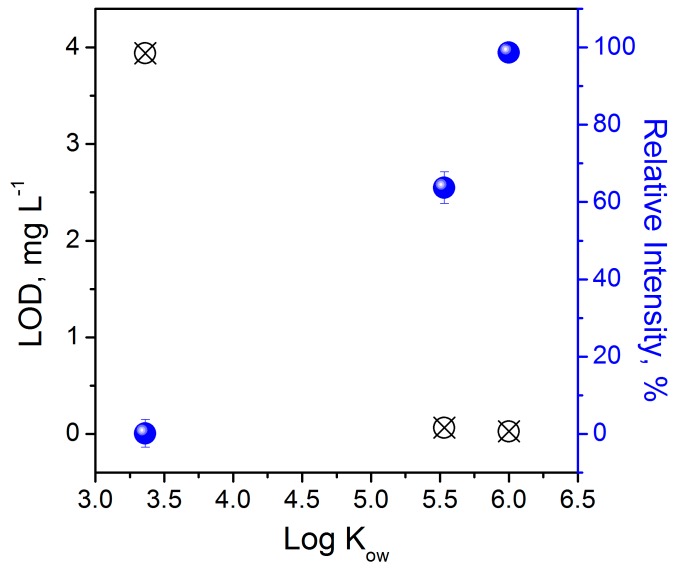
Correlation of the limit of detection (LOD) concentration (crossed circles) and the observed related surface-enhance Raman spectroscopy (SERS) intensities of the analytes in the mixed solution (blue circles) plotted as a function of the K_ow_ factor of the investigated compounds.

The results obtained on targeted pollutants in their individual solutions and in mixture provide important information on (i) the sensor effectiveness for PAH sensing and (ii) the sensor selectivity for BaP, FL, or NAP. First, the LOD concentrations for the investigated PAH compounds varied by two orders of magnitude (Table 3) between BaP and NAP. The correlation of the observed LOD values versus the log of the K_ow_ factor of the investigated analytes is given in Figure 6. This curve suggests that the hydrophobic coating of the sensor is more suitable for the preconcentration of highly non-polar PAH compounds.

This relationship is confirmed by the results obtained from the detection of pollutants in the mixed solution. Figure 6 shows the relative SERS intensity of the pollutant peaks calculated as follows:

(4) $$Relative\;Intensity\left(\%\right)=100\%*\frac{{I^{\prime }}_{Peak\;ofAnalyte}}{{I^{\prime }}_{Peak\;of\;DS-C_{10}H_{21}}}*\frac{I_{Peak\;of\;DS-C_{10}H_{21}}}{I_{Peak\;ofAnalyte}}$$

where *I*’ is the SERS intensity of the selected peaks measured in the mixed solution and *I* is the SERS intensity of the peaks measured for each individual PAH at the same concentration as that used in the mixed solution (0.75 mg L^−1^ for BaP and 5 mg L^−1^ for FL and NAP). The peaks used for this calculation are those used for the establishment of the calibration curves (the peak of DS-C_10_H_21_ at 1079 cm^−1^ and the peaks at 1239 cm^−1^, 1104 cm^−1^, and 1382 cm^−1^ for BaP, FL, and NAP, respectively). With this formula, the relative SERS intensity of the PAH signal obtained in the mixed solution was directly compared with the relative SERS intensity given by the calibration curve for the same pollutant concentration and it was then expressed as a percentage. For instance, for BaP, at a concentration of 0.75 mg L^−1^, the relative intensity of the peak at 1239 cm^−1^ measured in the calibration curve and in the mixed solution were found to be almost identical, leading to a calculated relative intensity of 98.7%. This value shows that the adsorption of BaP onto the nanosensor surface was not restricted by the presence of competitive analytes. The FL calibration curve indicates that at a concentration of 5 mg L^−1^, the adsorption equilibrium was reached with a maximum relative intensity of 0.22 ± 0.01. At the same concentration, FL in the mixed solution exhibited a relative intensity of 0.140 ± 0.012. This corresponds to 64% of the relative intensity reached with FL alone. The calculation of the NAP signal recorded in the mixed solution was more challenging. Due to the low intensity of the non-overlapping peak, it was not possible to quantify the intensity with the same formula. The approximate contribution of NAP was calculated indirectly. Since NAP and BaP both have peaks around 1382 cm^−1^, we subtracted the SERS spectra of BaP detection at 0.75 mg L^−1^ concentration from that measured in the mixed solution. The relative intensity of NAP was then found to be 0.14%.

The calculated relative intensities of the PAHs peaks in the SERS spectra obtained in the mixed solution confirmed that the strength of the interaction between the coating layer and the analyte have a strong influence on the preconcentration and thus on the detection of the analytes. Highly hydrophobic molecules having a high K_ow_ value will be easier to detect and will exhibit lower LOD. Moreover, the SERS nanosensor allowed the detection of BaP with a sensitivity comparable to the detection carried out using colloidal nanoparticles [81,82].

### 3.9. Surface Regeneration

**Figure 7:**
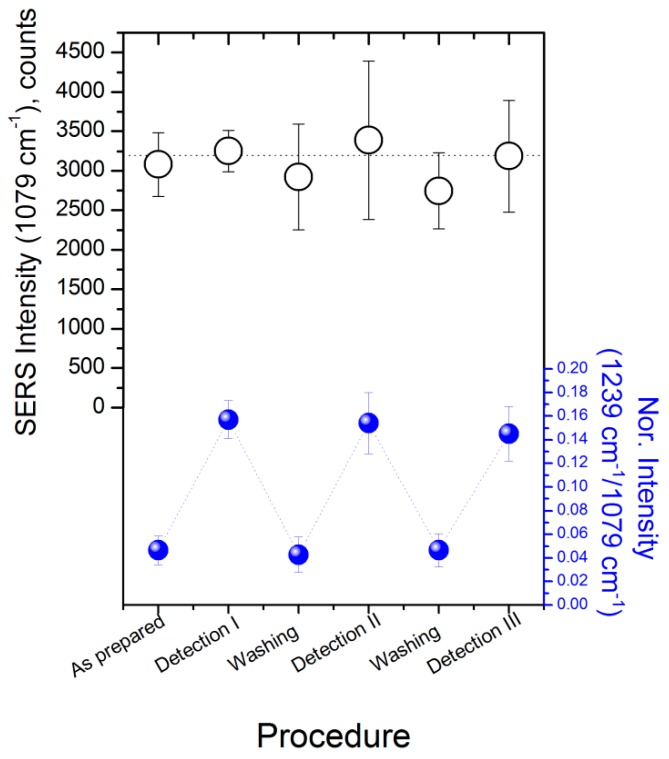
Surface-enhanced Raman spectroscopy (SERS) intensity variations for the peak at 1079 cm^−1^ of the diazonium salt (DS-C_10_H_21_) coating measured after each procedure (open black circles); SERS intensity ratios between the peaks at 1236 cm^−1^ and 1079 cm^−1^ for benzo[a]pyrene (BaP) and the DS-C_10_H_21_ coating, respectively (filled blue circles).

To demonstrate the reusability of the developed nanosensor, we performed regeneration experiments using a pure MeOH solution to remove the adsorbed PAHs from the sensor surface. For each step of regeneration, MeOH was injected and circulated in the fluidic cell for 30 min. To determine the impact of the surface regeneration step on the nanosensor performance, we screened for BaP at a concentration of 0.75 mg L^−1^. For this demonstration, BaP was selected for its strong interaction with the coated layers (as discussed above). Figure 7 summarizes the results of (i) SERS signal intensities for the peak at 1079 cm^−1^ assigned to the DS-C_10_H_21_ coating and (ii) the SERS intensity ratio between the peak of BaP at 1236 cm^−1^ and the peak of the DS-C_10_H_21_ coating for each experimental step (detection and washing). The surface regeneration process did not affect the stability of the functionalization layer, and the average intensity of the peak was found to be nearly constant with a variation of 11% between the different experimental steps. Moreover, several cycles of BaP screening show highly reproducible SERS signals of the analyte with an average intensity ratio of 0.152 with a standard deviation lower than 5%. This result demonstrates that the nanosensor surface can be washed and therefore reused several times without affecting the detection efficiency.

### 3.10. Detection Reproducibility and Repeatability

The high reproducibility and repeatability of the SERS signal of the developed nanosensor is of primary importance for its application to quantitative analyte detection. Statistical analysis was carried out to estimate the repeatability of the SERS signal intensity between various spots on the substrate as well as to define reproducibility between two functionalized nanosensors. For this, SERS spectra of BaP at a concentration of 0.75 mg L^−1^ were recorded on six individual spots for two substrates. By comparing the calculated relative intensity ratios between the signal of BaP and the signal of the coating (I_1236 cm_^−1^/I_1079 cm_^−1^), we attained very reproducible targeted pollutant detection. Calculated average values were 0.147 and 0.152 for different substrates with a coefficient of variation of 15% and 13%, respectively. These results indicate that commercially available Wavelet SERS substrates functionalized with a diazonium salt can be considered as a repeatable and reproducible SERS solution. To further demonstrate the suitability of our diazonium salt-SERS solution, we compared it with a popular commercial SERS substrate, Klarite, for which a coefficient of variation of 45% has been reported from the measurement of benzenethiol grafted to the surface [83]. Moreover, the low coefficient of variation observed from spot-to-spot measurements suggests that DS-C_10_H_21_ diazonium-salt-based surface functionalization offers robust and homogeneous surface coverage.

## 4. Conclusions

Here, we demonstrated a novel approach for the decoration of a SERS nanosensor for the reversible and reproducible detection of non-polar pollutants. Diazonium salt (DS-C_10_H_21_) was tested for substrate surface functionalization and for subsequent preconcentration of the targeted compounds. Spontaneous DS-C_10_H_21_ layer formation was found to be highly reproducible under the operating conditions. During this conceptual study, we demonstrated the detection of BaP, FL, and NAP with LOD values of 0.026 mg L^−1^ (0.1 µmol L^−1^), 0.064 mg L^−1^ (0.32 µmol L^−1^), and 3.94 mg L^−1^ (31 µmol L^−1^), respectively. The LOD concentrations of the detected compounds were strongly correlated with their physicochemical properties. The observed selectivity of the coated nanosensor favoring highly non-polar molecules such as BaP suggests that the preconcentration of aromatic pollutants can be optimized by varying the nature of the aryldiazonium salt used for surface functionalization. 

The level of BaP detection obtained in this study is comparable with that documented in the literature using colloidal nanoparticles. Given that the major drawback of colloidal nanoparticles’ application for pollutant monitoring is signal reproducibility, our proposed nanosensor functionalized with diazonium salt holds great promise for applications to in situ measurements due to its demonstrated sensitivity, reproducibility, repeatability, and reusability.

## Figures and Tables

**Table 1:** Physical properties of the selected targeted pollutants [70].

Polycyclic Aromatic Hydrocarbon	Structure	Solubility in Water (mg L^−1^)	K_ow_
Benzo[a]pyrene (BaP)	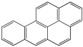	0.0038 at 25 °C	10^6^
Fluoranthene (FL)	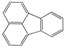	0.26 at 25 °C	3.4 × 10^5^
Naphthalene (NAP)	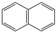	32	2.3 × 10^3^

**Table 2:** Positions in cm^−1^ of the main polycyclic aromatic hydrocarbon (PAH) bands observed in the Raman and SERS spectra (see [77,78] for assignments).

Benzo[a]Pyrene (BaP)		Fluoranthene (FL)		Naphthalene (NAP)	
Raman	SERS	Raman	SERS	Raman	SERS
615	615	562	562	509	511
1215	1215	671	672	762	762
1236	1239	803	803	1021	1021
1345	1345	1018	1018	1382	1382
1389	1385	1104	1104	1577	1577
1623	1623	1269	1270		
		1612	1612		

**Table 3:** Summarized limit of detection (LOD) and limit of quantitation (LOQ) values for all targeted pollutants.

Polycyclic Aromatic Hydrocarbon	LOD		LOQ	
	(mg L^−1^)	(mol L^−1^)	(mg L^−1^)	(mol L^−1^)
Benzo[a]pyrene (BaP)	0.026	10^−7^	0.087	3.4 × 10^−7^
Fluoranthene (FL)	0.064	3.2 × 10^−7^	0.214	1.1 × 10^−6^
Naphthalene (NAP)	3.9	3 .0× 10^−5^	13	1.0 × 10^−4^

## Supplementary Materials

The Supplementary Materials containing Figures S1–S5 are available online at http://www.mdpi.com/1424-8220/17/6/1198/s1.
