# Supplementary material for: Diazonium Salt-Based Surface-Enhanced Raman Spectroscopy Nanosensor: Detection and Quantitation of Aromatic Hydrocarbons in Water Samples
Source: Sensors (Basel). 2017 May 24;17(6):1198. doi: 10.3390/s17061198 (PMC5492876; doi:10.3390/s17061198)
Supplement: Supplementary file 1 [file sensors-17-01198-s001.pdf]

Supplementary Information

Diazonium Salt-Based Surface-Enhanced Raman Spectroscopy Nanosensor: Detection and Quantitation of Aromatic Hydrocarbons in Water Samples

Inga Tijunelyte [1], Stéphanie Betelu [2], Jonathan Moreau [3], Ioannis Ignatiadis [2], Catherine Berho [2], Nathalie Lidgi-Guigui [1], Erwann Guénin [1], Catalina David [4], Sébastien Vergnole [4], Emmanuel Rinnert [3], Marc Lamy de la Chapelle [1]

[1] CSPBAT Laboratory, UMR 7244, UFR SMBH, University of Paris 13, Sorbonne Paris Cite, 93017 Bobigny, France; inga.tijunelyte@univ-paris13.fr (I.T.); nathalie.lidgi-guigui@univ-paris13.fr (N.L-G.); guenin@univ-paris13.fr (E.G); marc.lamydelachapelle@univ-paris13 (M.L.C)

[2] BRGM, F-45060 Orléans Cedex 02, France; s.betelu@brgm.fr (S.B.); i.ignatiadis@brgm.fr (I.I.); C.Berho@brgm.fr (C.B.)

[3] IFREMER, Brittany Center, Detection, Sensors and Measurements Laboratory, CS10070, 29280 Plouzané, France; Jonathan.Moreau@ifremer.fr (J.M.); Emmanuel.Rinnert@ifremer.fr (E.R.)

[4] HORIBA Jobin Yvon SAS, 59650 Villeneuve d'Ascq, France; Catalina.DAVID@horiba.com (C.D.); sebastien.vergnole@horiba.com (S.V.)

Correspondence: marc.lamydelachapelle@univ-paris13.fr; Tel.: +33-1-48-38-7691, s.betelu@brgm.fr ; Tel.: +33-2-38-64-3268

KEYWORDS:

polynuclear aromatic hydrocarbon (PAH); surface-enhanced Raman spectroscopy (SERS); nanosensor; diazonium salt; surface functionalization; detection.

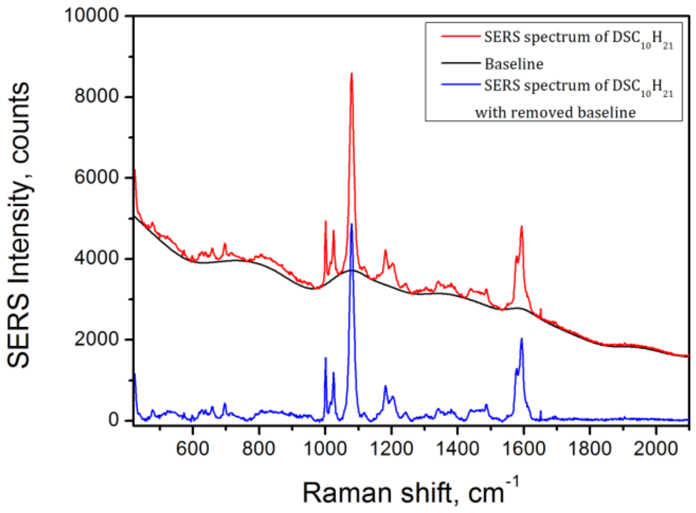

Figure S1: Example of the baseline correction procedure used in this study.

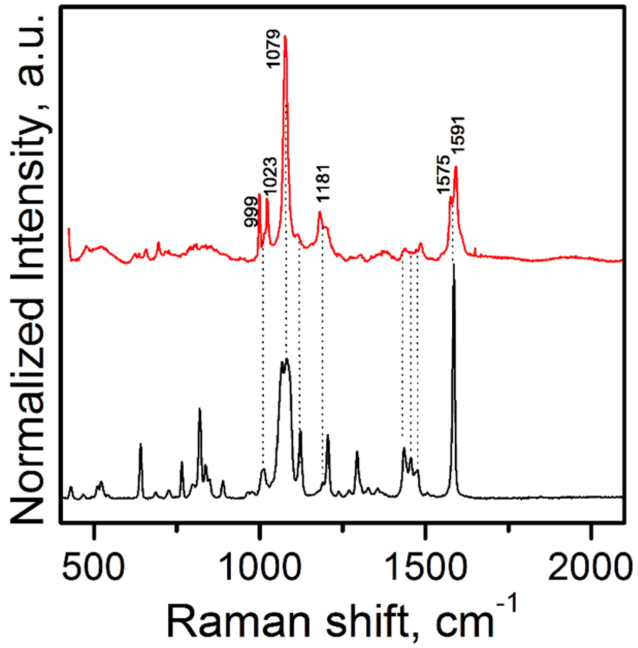

Figure S2: Comparison of Raman signatures (back spectrum) of selected aryldiazonium salt DS-$C_{10}H_{21}$ with SERS spectrum (in red) of their grafted layers.

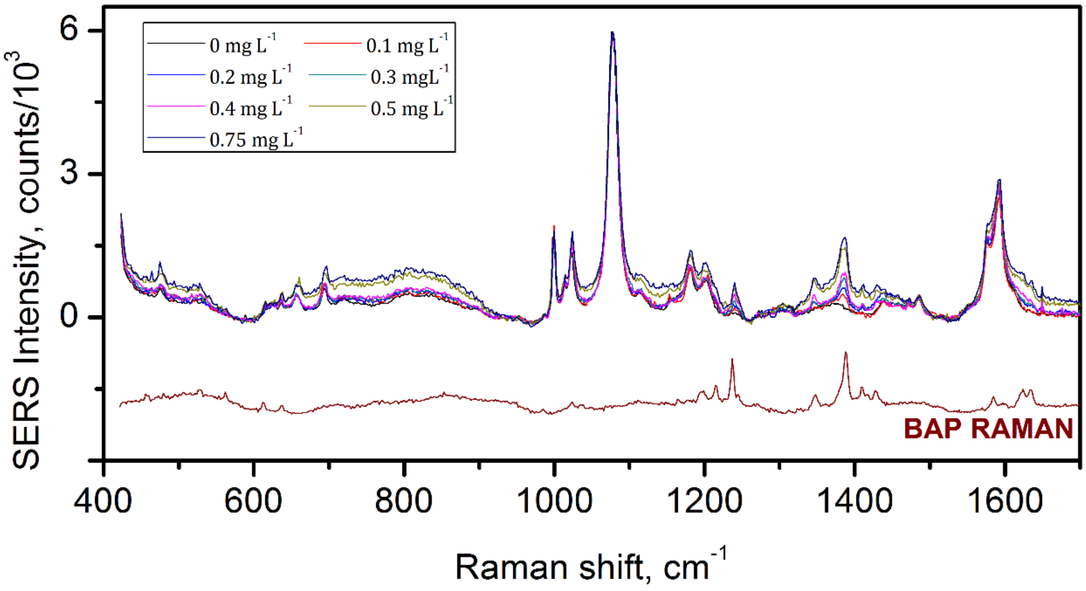

Figure S3: Detection of benzo[a]pyrene using a DS-$C_{10}H_{21}$ diazonium-salt-based nanosensor. Representation of the complete spectral range.

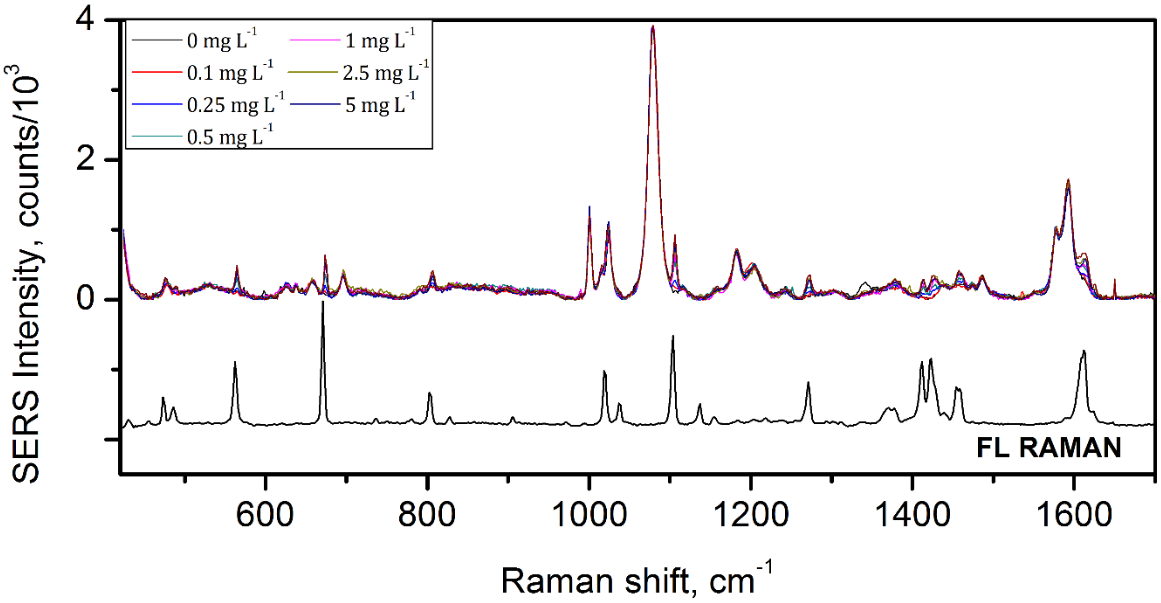

Figure S4: Detection of fluoranthene (FL) using a DS-$C_{10}H_{21}$ diazonium-salt-based nanosensor. Representation of the complete spectral range.

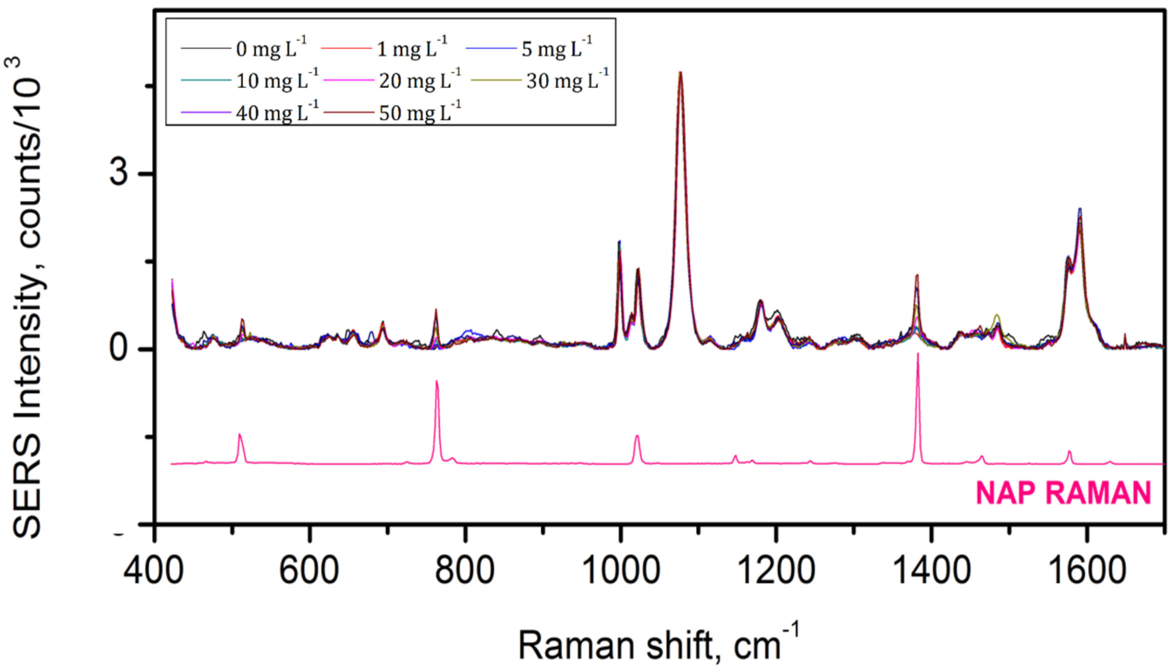

Figure S5: Detection of naphthalene (NAP) using a DS-$C_{10}H_{21}$ diazonium-salt-based nanosensor. Representation of the complete spectral range.
